# Parametrically Amplified Low-Power MEMS Capacitive Humidity Sensor

**DOI:** 10.3390/s19183954

**Published:** 2019-09-13

**Authors:** Rugved Likhite, Aishwaryadev Banerjee, Apratim Majumder, Mohit Karkhanis, Hanseup Kim, Carlos H. Mastrangelo

**Affiliations:** Department of Electrical and Computer Engineering, University of Utah, Salt Lake City, UT 84112, USA; rugved.likhite@utah.edu (R.L.); aishwaryadev.banerjee@utah.edu (A.B.); apratim.majumder@utah.edu (A.M.); mohit.karkhanis@utah.edu (M.K.); hanseup@ece.utah.edu (H.K.)

**Keywords:** humidity sensor, low-power sensors, MEMS, parametric amplification, spring softening

## Abstract

We present the design, fabrication, and response of a polymer-based Laterally Amplified Chemo-Mechanical (LACM) humidity sensor based on mechanical leveraging and parametric amplification. The device consists of a sense cantilever asymmetrically patterned with a polymer and flanked by two stationary electrodes on the sides. When exposed to a humidity change, the polymer swells after absorbing the analyte and causes the central cantilever to bend laterally towards one side, causing a change in the measured capacitance. The device features an intrinsic gain due to parametric amplification resulting in an enhanced signal-to-noise ratio (SNR). Eleven-fold magnification in sensor response was observed via voltage biasing of the side electrodes without the use of conventional electronic amplifiers. The sensor showed a repeatable and recoverable capacitance change of 11% when exposed to a change in relative humidity from 25–85%. The dynamic characterization of the device also revealed a response time of ~1 s and demonstrated a competitive response with respect to a commercially available reference chip.

## 1. Introduction

In recent decades, miniaturized humidity sensors have been realized using various transduction methods [1,2,3,4,5,6,7] for a wide range of applications such as improving indoor comfort in homes and automobiles, humidity monitoring in semiconductor processing facilities [8], food processing industries [9], medical facilities [10] and, Internet-of-Things (IoT) based frameworks [11]. The push for the need for low-power chemical sensors [12,13,14] has been quite strong due to the growing importance of IoT sensor nodes across the world.

The most commonly used humidity sensor for the applications mentioned above is the capacitive sensor, which is used in nearly 75% of the cases [3] as nearly zero DC current flows through them making them extremely low power consuming devices. These devices measure the change in capacitance caused by variations in dielectric properties or thickness of a sensing layer sandwiched between two parallel plates [15,16] when exposed to humidity. Sensors based on measuring the deflection of a microcantilever coated with a sensing polymer have also been demonstrated [17,18]. These sensors show a linear behavior, are easy to batch fabricate, and most importantly, consume nearly zero DC power. However, their Signal-to-Noise Ratio (SNR) is usually limited by electronic noise as their sense capacitance can be small in comparison to the surrounding parasitics due to their small size [19]. Amplifiers are typically used in conjunction with such sensors to obtain a stable output, which further adds to the total noise of the system and limits the SNR.

The sensitivity of capacitive hygrometers can be significantly improved if these devices have an intrinsic gain, thus reducing the dependence on noisy electronic amplifiers. This can be achieved with mechanical leveraging and parametric amplification. An example of a mechanically leveraged structure is a microcantilever device coated on one side with a sensing layer. Highly sensitive microcantilever-based sensors have been reported previously for detecting gases [18,20,21], DNA hybridization [22,23], and toxic chemical warfare agents [24]. In these devices, the exposure of the sensing film to an analyte or temperature change generates surface stress that induces bending of the free-standing cantilever either due to a reduction in interfacial surface energy or swelling of the sensing layer. High signal to noise ratio can be realized in such devices using parametric amplification while maintaining small transducer size and low power consumption by exploiting the voltage induced lateral instability in Micro-Electro-Mechanical Systems (MEMS) devices to magnify their displacement to capacitance sensitivity. This technique has been previously reported to improve the performance of a MEMS magnetometer [19], gyroscope [25], hair-flow sensor [26], and vapor sensors [27,28,29]. Unlike electronic amplification, parametric amplification has an inherent advantage of providing higher sensitivity in MEMS sensor systems, as it amplifies the sensor signal without adding any extra electronic noise to the circuit.

In this paper, we report the design, fabrication, and testing of a new type of low power, batch-fabricatable, parametrically amplified, microcantilever-based humidity sensor with improved sensitivity. This article expands on a proof of concept presented earlier [29]. Extensive characterization of the sensor response has been presented in this article, along with dynamic response testing and comparison to a commercially available sensor. Additionally, a relevant analysis of the sensor output and a mathematical model describing the sensor action is also presented, along with a study on the sorption kinetics of the device.

## 2. Device Structure and Operation

**Figure 1:**
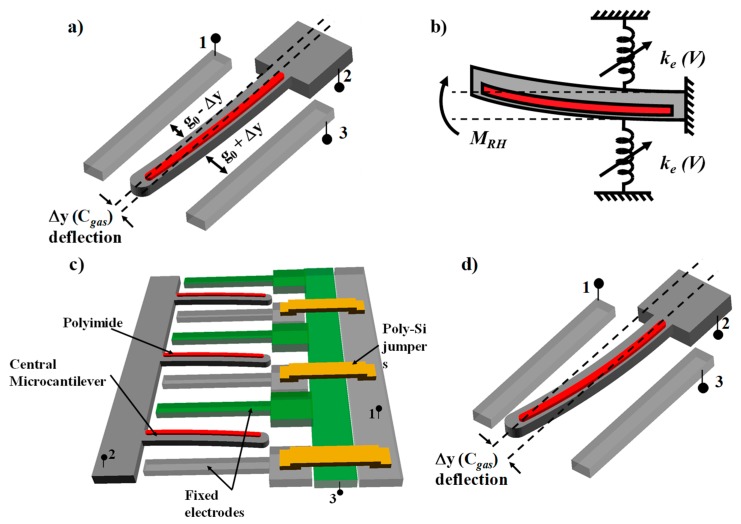
(**a**) Unit cell of the LACM humidity sensor. (**b**) Mechanical equivalent of the device when subjected to a change in relative humidity. (**c**) Array structure of parallel LACM unit cells. (**d**) Use of parametric amplification to magnify the deflection sensitivity of the central microcantilever.

A unit cell of the LACM sensor is shown in Figure 1a. The device consists of a suspended microcantilever beam (electrode 2) asymmetrically coated on top with a sensing polymer (polyimide) and flanked on the sides by two stationary electrodes (1 and 3). When the device is exposed to an analyte vapor, the polyimide absorbs the gas and swells. This exerts a bending moment, M_RH_, on the structural beam causing it to deflect to one side (Figure 1b). Unlike conventional polymer-based microcantilever sensors, which measure the out-of-plane deflection of the cantilever [30], the LACM sensor measures the in-plane deflection of the sensing cantilever by forming two parallel plate variable capacitors between the central finger and the adjacent electrodes, as shown below. Our devices are appropriately designed to make the out-of-plane stiffness much higher than the in-plane spring constant. A planar design allows multiple unit cell structures to be united in parallel to increase the total output signal from the sensor (Figure 1c) while maintaining compatibility with conventional CMOS processes suitable for low power, high sensitivity water vapor sensors for application in IoT frameworks.

Furthermore, parametric amplification of the output signal is achieved by applying a symmetric DC bias voltage to both the flanking electrodes (1 and 3) with respect to the central suspended electrode (2) to improve the vapor-concentration to displacement sensitivity of the device (Figure 1d). In this work, the single side capacitance measurements for the device have been reported. Theoretically, the sensor performance can be further improved by measuring a differential capacitance between the two sides while also eliminating common mode parasitics.

### 2.1. Electrostatic Spring Softening

Parametric amplification induced spring softening in the mechanical domain has been extensively studied to tune the resonant frequency of the MEMS structures [31,32,33] and to produce large-amplitude deflections in microstructures [26,34]. In the LACM sensor, when a DC bias voltage is applied to the electrodes 1 and 3 with respect to electrode 2, the non-linearity of the electrostatic forces acting on the central cantilever beam results in the reduction of the effective spring constant of the central cantilever. Mathematically, electrostatic spring softening of micromechanical systems can be observed by minimizing the total energy (*U_T_*) function of the system:

(1) $$U_{T}=U_{EL}+U_{M}$$

where *U_EL_* is the electrostatic energy stored in the capacitors of the system and *U_M_* is the mechanical energy stored in the deformed microcantilever beam. For a microcantilever beam deflecting laterally between two electrodes, as shown in Figure 1, the respective energies can be written as:

(2) $$U_{EL}=\frac{-\varepsilon AV_{b}^{2}}{2}\left(\frac{1}{g_{0}+\Delta y}+\frac{1}{g_{0}-\Delta y}\right)$$

(3) $$U_{M}=\;\frac{k_{o}\Delta y^{2}}{2}$$

where *g*_0_ is the initial gap between the electrodes, Δ*y* is the tip deflection of the central beam due to absorption induced polymer swelling, *V_b_* is the applied DC bias voltage, *A* is the overlap area of the capacitor, *ε* is the permittivity, and *k_o_* is the lateral spring constant of the central finger when no bias is applied. For $\frac{\Delta y}{g_{0}}\ll 1$, the total energy of the system can be written as:

(4) $$U_{T}=\frac{1}{2}k_{o}\Delta y^{2}-\left[\frac{\varepsilon AV_{b}^{2}}{g_{0}}\right]\left(\frac{1}{1-{\left(\frac{\Delta y}{g_{0}}\right)}^{2}}\right)$$

(5) $$U_{T}=\frac{1}{2}k_{o}\left[\Delta y^{2}-\left(\frac{2\varepsilon AV_{b}^{2}}{k_{o}g_{0}}\right)\left(1+{\left(\frac{\Delta y}{g_{0}}\right)}^{2}-{\left(\frac{\Delta y}{g_{0}}\right)}^{4}+\;\ldots \ldots \right)\right]$$

(6) $$U_{T}≅\frac{1}{2}k_{o}\underset{k\left(M\right)}{\underbrace{\left[1-\left(\frac{2\varepsilon AV_{b}^{2}}{k_{o}g_{0}^{3}}\right)\right]}}\Delta y^{2}+h.o.t$$

For $V_{p}=\;\sqrt{\frac{k_{o}g_{0}^{3}}{2\varepsilon A}}$, which is the symmetrically-biased pull-in voltage for the structure, the effective softened spring constant *k*(*M*), can be written as:

(7) $$k\left(M\right)=\frac{k_{o}}{M}\;where,\;M=\frac{1}{\left(1-\frac{V_{b}^{2}}{V_{p}^{2}}\right)}\;$$

**Figure 2:**
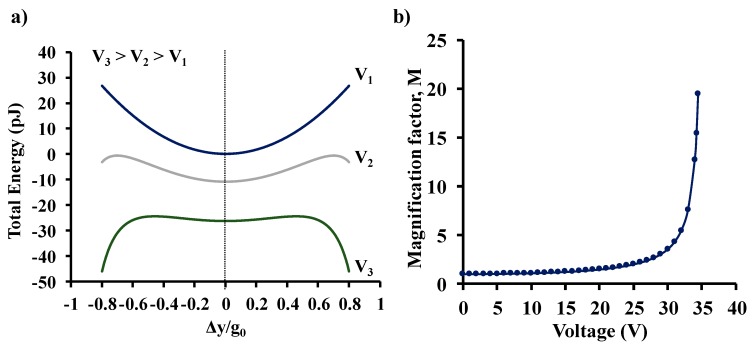
Schematic of the (**a**) total energy of the system during parametric amplification, and (**b**) magnification factor *M* as a function of the applied voltage.

For example, Figure 2a shows the total energy of a system with *k_o_* = 3.7 Nm^−1^ and *V_p_* = 35.4 V without DC bias (*V*_1_ = 0 V) and, when DC voltage bias (*V*_2_ = 18 V and *V*_3_ = 28 V) is applied to induce parametric amplification, as a function of normalized beam deflection. As the magnitude of the applied voltage bias is increased, the system becomes progressively unstable due to the shallowing of the local energy minima. Since $k\left(M\right)<k_{o}$, the output signal is magnified by a voltage-dependent magnification factor, M, as shown in Figure 2b. Note that the above derivation is an approximation as it assumes Δ*y* to be the uniform change in the gap across the entire length of the beam. Though parametric amplification can be used to obtain signal magnification, it is important to note that operating very close to the pull-in voltage (very high gains) would require additional voltage stabilization electronics to prevent the beam from latching sideways. Additionally, inducing higher degrees of spring softening does limit the operational range of the sensor because the range of stable deflections for the central beam before pull-in decreases, as voltage bias is increased. 

### 2.2. Noise Analysis and Signal-to-Noise Ratio

**Figure 3:**
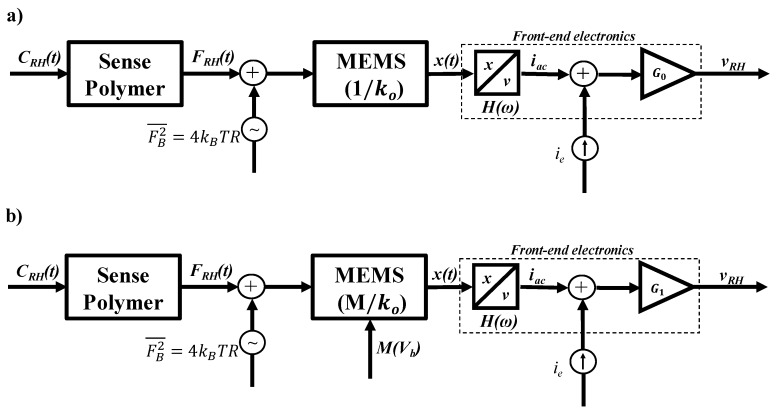
(**a**) Schematic of the sensor system at zero bias with default spring constant *k_o_* (**b**) with parametric amplification (near-Brownian noise is limited).

The noise of the system originates from both the mechanical-thermal noise [35,36] of the deflecting beam and the noise of the C/V converter operational amplifiers [37]. In our device, we convert the stress caused by the RH absorption (*F_RH_*) into a mechanical deflection, which translates into a variable, and RH dependent capacitance, mediated through the beam spring constant *k*(*M*) = *k_o_*/*M*, where *M* is the bias-dependent magnification factor. We use high-gain op-amps to amplify and read the sensor capacitance [38], as illustrated in the schematic of Figure 3a below.

The capacitance change indicative of the vapor concentration signal is first converted to an AC current with $i_{ac}=j\omega C\left(RH\right)\cdot V_{o}$, where *V_o_* is the amplitude of a sinusoidal AC voltage of angular frequency *ω* placed across the capacitor. The AC current is finally converted to an output voltage using a high-gain op-amp of transconductance *G_o_*. The noise of the system is calculated by the introduction of two noise sources: (1) a mechanical noise acting on the beam with spectral density [35,39]:

(8) $$F_{B}=\;\sqrt{4k_{B}TR}\;\left[N/\surd Hz\right]$$

which is only dependent on the Boltzmann constant *k_B_*, temperature T, and viscous damping coefficient R of the system, as described previously in a viscous damping environment (air) and at low pressures [36]. Therefore, the noise force on the MEMS device is completely independent of the bias induced spring softening used in the LACM sensor. (2) The electronic noise current source $\bar{i_{e}^{2\;}}$, which is introduced at the input of the transconductance amplifier. The output noise for this system is thus:

(9) $$\bar{v_{o}^{2\;}\left(k_{o}\right)}=\;G_{o}^{2}\left(\omega^{2}V_{o}^{2}\;F^{2}\frac{\bar{F_{B}^{2\;}}}{k{\left(M\right)}^{2}}+\bar{i_{e}^{2\;}}\right)=\;G_{o}^{2}\left(\omega^{2}V_{o}^{2}\;F^{2}\;\frac{\bar{F_{B}^{2\;}}}{k_{o}^{2}}+\bar{i_{e}^{2\;}}\right)=G_{o}^{2}\left(H^{2}\left(\omega \right)\cdot \frac{\bar{F_{B}^{2\;}}}{k_{o}^{2}}+\bar{i_{e}^{2\;}}\right)$$

where

(10) $$H^{2}\left(\omega \right)=\;\omega^{2}V_{o}^{2}\;F^{2}\;,\;F^{2}=\left(\frac{dC}{dF_{B}}\;\right){\;}^{2}\;$$

Since the deterministic signal $v_{RH}^{2}=\;H^{2}\left(\omega \right)\cdot \frac{F_{RH}^{2}}{k_{o}^{2}}\;\cdot G_{o}^{2}$, where *F_RH_* is the equivalent RH-driven force, the signal to noise ratio is thus given by:

(11) $$SNR_{o}=\frac{\frac{F_{RH}}{k_{o}}\cdot H\left(\omega \right)}{\sqrt{\frac{F_{B}^{2}}{k_{o}^{2}}\cdot H^{2}\left(\omega \right)+i_{e}^{2}}}=\frac{\left(\frac{F_{RH}}{F_{B}}\right)}{\sqrt{1+\left(\frac{k_{o}^{2}\;}{H^{2}\left(\omega \right)\cdot F_{B}^{2\;}}\right)\cdot i_{e}^{2}}}.\ldots =\frac{SNR_{max}}{\sqrt{1+\left(\frac{k_{o}^{2}\;}{H^{2}\left(\omega \right)\cdot F_{B}^{2\;}}\right)\cdot i_{e}^{2}}}$$

It is evident that the SNR is independent of transconductance *G_o_*. Equation (11) also tells us that any electromechanical effect that lowers *k_o_* will result in a higher SNR. In our device, this can be achieved with the bias-induced spring softening gain *M*, as shown in the schematic of Figure 3b, such that:

(12) $$SNR\left(M\right)=\;\frac{SNR_{max}}{\sqrt{1\;+\frac{\left({\left(\frac{SNR_{max}}{SNR_{o}}\right)}^{2}-1\right)}{M^{2}}}}$$

The SNR is improved for *M* > 1 essentially because the spring softening effect gain is noiseless, thus moving the electronic noise closer to the amplified output.

### 2.3. Sensor Response Model

The LACM sensor is essentially a humidity-dependent variable and parallel-plate capacitor. When the device is exposed to humidity, the sensing polymer swells after absorbing the water vapor. Since the polymer is asymmetrically patterned and constrained to the silicon cantilever beam, the swelling generates surface stress [40], which results in the bending of the cantilever beam towards one side. This results in a change in the measured capacitance of the device. The amount of cantilever bending and, therefore, the capacitance change is directly proportional to the swelled induced surface stress and inversely proportional to the effective spring constant of the device given by Equation (7). This can be mathematically described by a modified form of Stoney’s equation as given by Godin et al. [41].

For small displacements of the central cantilever beam, the normalized change in capacitance can be written as:

(13) $$\frac{\Delta C}{C}=\left(A_{o}\right)\left[\frac{\left(1-\nu \right)l^{2}}{g_{0}E_{Si}w^{2}}\cdot \frac{1}{\left(1-\frac{V^{2}}{V_{p}^{2}}\right)}\cdot \beta_{p}\cdot C_{RH}\right]+B_{o}$$

where *ν* = Poisson’s ratio of Silicon, *l* = length of central cantilever beam, *g*_0_ = initial air-gap between electrodes, *E_Si_* = Young’s Modulus of Silicon, *w* = width of central cantilever beam, *V* = applied voltage bias to induce spring softening, *V_p_* = symmetrically-biased pull-in voltage of beam, *β_p_* = fitting parameter proportional to the swelling-induced surface stress generated by the polyimide, and *C_RH_* is the relative humidity of the chamber. *A_o_* and *B_o_* are dimensionless fitting parameters.

### 2.4. 2-Level Electrical Interconnects

The laterally deflecting and planar design of the LACM sensor allows the connection of multiple unit cells in parallel in order to further increase the output of the sensor. Since each microcantilever is flanked on the two sides by electrically isolated anchored electrodes, it is necessary to have a 2-level electrical connection arrangement in the device. In the LACM sensor array, this is done by fabricating jumpers out of doped poly-Si, as shown in Figure 1c. The detailed fabrication procedure is described in the following sections. The jumper arrangement eliminates the needs for wire bonds to connect multiple devices, thus keeping the fabrication process simple.

## 3. Fabrication and Imaging

### 3.1. Device Fabrication

**Figure 4:**
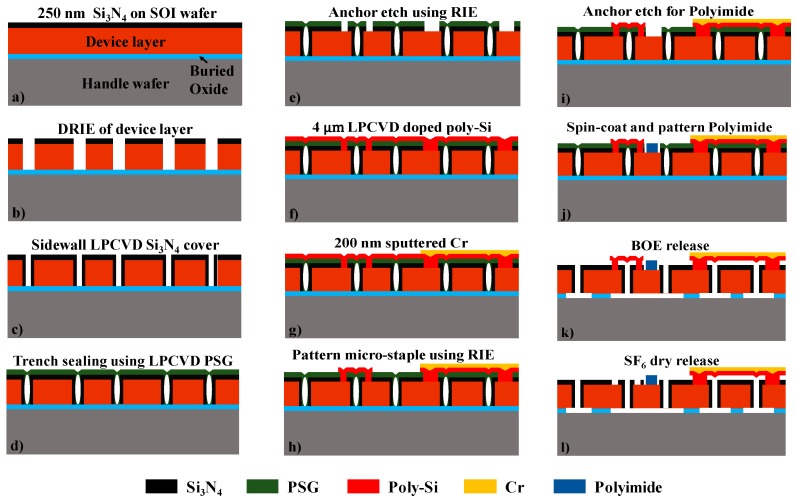
Simplified fabrication procedure of the device.

Figure 4a–l shows a simplified fabrication procedure of the device. The process starts by depositing 250 nm of low-stress silicon nitride using a Low Pressure Chemical Vapor Deposition (LPCVD) process over Silicon-on-Insulator (SOI) wafers with a 30 µm thick device layer and a 2 µm thick buried oxide layer, as shown in Figure 4a. The nitride layer is patterned using conventional UV photolithography width and then etched using CF_4_/O_2_ RIE. The device layer of the SOI wafer is then etched using Deep Reactive Ion Etching (DRIE) to form the fingers (Figure 4b). A low-frequency RF source (380 kHz) is used for this process to avoid footing and prevent premature release of the device. The photoresist is then removed using acetone, and a pre-furnace clean is performed. A 100 nm thin layer of LPCVD silicon nitride is then deposited on the wafer, and a blanket CF_4_/O_2_ RIE etch is done. This ensures that the nitride remains only on the sidewalls of the etched fingers (Figure 4c). A 4 µm thick layer of sacrificial LPCVD Phosphosilicate glass (PSG) is then deposited on the wafer and annealed in an N_2_ environment at 1050 °C to reflow the PSG. The thickness of the PSG is then reduced to ~2 µm using a blanket RIE etch on the wafer. This deposition-reflow and etch back process is repeated until the etched gaps between different fingers are entirely sealed (Figure 4d) due to the cusping effect in an LPCVD process, thus allowing further processing of the wafer [42,43]. The sacrificial PSG and the underlying nitride are then patterned using photolithography and RIE to create anchors (Figure 4e) for poly-Si jumpers and the anti-stiction micro-staple pins. A 4 µm thick layer of poly-Si is then deposited using the LPCVD process. This layer is then doped using phosphorus solid-source doping and annealed at 1050 °C for 2 h (Figure 4f). A 200 nm thick layer of Cr is then deposited over the wafer using DC-sputtering and patterned using a wet etchant to form the metal contact and jumpers (Figure 4g). The wafer is the then cleaned, and the anti-stiction features are patterned (Figure 4h). This step also forms the poly-Si jumpers and contact pads, with the previously patterned Cr metal acting as an etch mask. The PSG on the central finger is then patterned and etched to create windows (Figure 4i) to allow deposition and anchoring of the sensing polymer to the device. We use HD-4104 polyimide [44] as a water vapor sensing material. The polyimide is spin-coated on the sample at 3000 rpm and soft-baked at 90 °C for 2 min, followed by 100 °C for 2 min. The polymer is then cured at 300 °C in an oven with N_2_ environment for 3 h. A commercially available Polyimide adhesion promoter, VM-651, is applied to the sample before spin-coating to improve the adhesion of the polyimide to the substrate and to prevent any delamination during the Buffered Oxide Etch (BOE) release procedure. The obtained thickness of the polyimide was ~3.35 µm, which was measured using a Tencor P-10 Profilometer. A 200 nm thick Al layer is then sputtered on the polyimide and then patterned asymmetrically on the central finger using wet etching to act as a hard mask. The polyimide is then etched using O_2_ plasma (Figure 4j) in an Oxford 100 ICP etcher to obtain a 7 µm wide polymer patch. The devices are then diced, and the chips are released in BOE for 160 min with constant stirring (Figure 4k). The etching time was determined by running a few trial samples for different etch times to confirm device release and determine the undercut rate. The chips are finally rinsed thoroughly in DI water followed by methanol and allowed to air dry.

### 3.2. Stiction Suppression

Stiction is one of the main modes of failure in MEMS devices [45]. Device failure due to stiction occurs when suspended MEMS structures, such as cantilevers, plates, or beams, adhere to the substrate or adjacent features due to lack of sufficient restoring force when subjected to strong capillary forces. Typically, capillary forces arise during the device fabrication and cleaning due to the surface tension of water when the sample is allowed to dry. Various methods have been previously used to prevent stiction due to surface tension [45,46,47]. In the LACM device, a different method which utilizes ‘micro staple-pins’ that hold the released cantilever in place during a wet release procedure, has been utilized. The staples are made out of thin poly-Si that can be dry etched, thus eliminating the need for complex and expensive anti-stiction processes.

A very short SF_6_ etch is finally performed on the devices to etch away the anti-stiction micro-staples and release the fingers (Figure 4l). Any unwanted etching on the side wall of the fingers is prevented by the thin Si_3_N_4_ film that is deposited over the finger before PSG sealing.

### 3.3. Imaging

**Figure 5:**
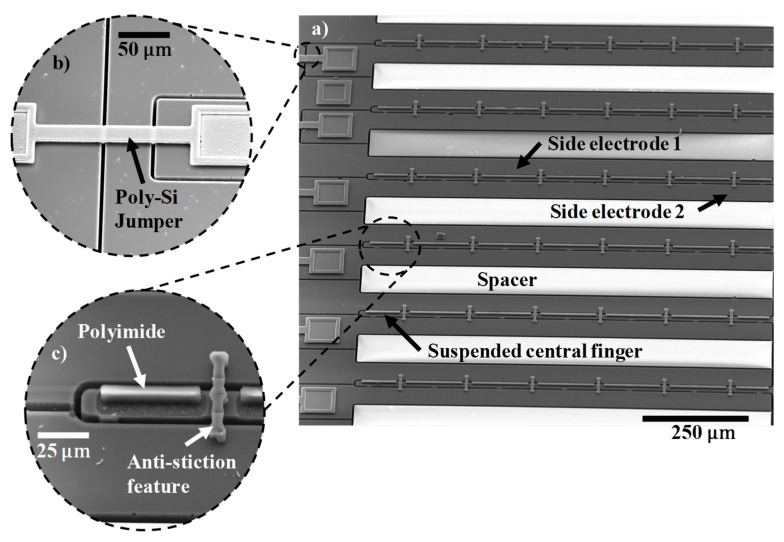
(**a**) LACM sensor array; (**b**) zoomed-in image of a poly-Si jumper over etched trenches acting as the second level of electrical connections; (**c**) magnified view of a suspended central finger with asymmetrically patterned polyimide and anti-stiction micro-staple holder.

High-resolution Scanning Electron Microscope (SEM) imaging of the device was done on an FEI Quanta 600 SEM at an accelerating voltage of 20.0 kV to verify the fabricated device structure, as shown in Figure 5a–c. Figure 5a shows the fabricated device array with the poly-Si jumpers acting as the second level of electrical connections, which are shown in the zoomed in image (Figure 5b). Only the central finger is released during the timed BOE wet etch, as the flanking electrodes are much wider and, therefore, stay anchored. Zoomed-in image of the central finger is visible in Figure 5c, showing the asymmetrically patterned polyimide on the movable beam and the anti-stiction micro-staple pins clamping the central finger to the side electrodes after wet release. The fabricated devices were 900 µm long, and the suspended beams were 13.3 µm wide. The air-gap measured between the suspended beam and the side electrode was 4.75 µm. The symmetrically-biased pull-in voltage was calculated to be ~29.5 V. The fabricated device had 15 unit cells connected in a parallel circuit.

## 4. Testing and Characterization

### 4.1. Test Setup

The sensor electrical testing was done at a probe station enclosed in a metallic box to create a localized environment for vapor testing. The enclosing box was grounded to reduce outside interference and noise during measurement. The device capacitance was measured using a Keithley 4200A-SCS CVU that was connected to the probe station at 1 MHz frequency using a 30 mV AC signal. The base capacitance of the device was measured to be 270 fF. The chamber was flushed with N_2_ gas before testing, and a commercial humidifier that was placed outside was used to humidify the chamber. The relative humidity (RH) of the chamber was monitored using a commercially available BME-280 [48] chip connected to an Arduino Uno board, which reported the chamber humidity to a computer. Dehumidification of the chamber was done by purging the test chamber with N_2_ while evacuating the chamber using an in-house vacuum line.

### 4.2. Sensor Action and Humidity Response

**Figure 6:**
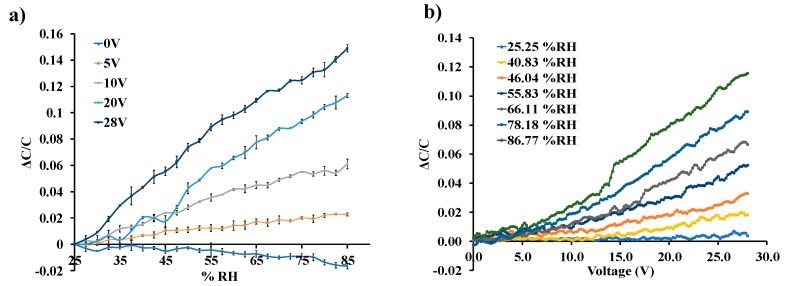
(**a**) Sensor response to varying %RH levels at different DC bias voltage; (**b**) sensor response to a varying bias voltage at different %RH levels.

**Figure 7:**
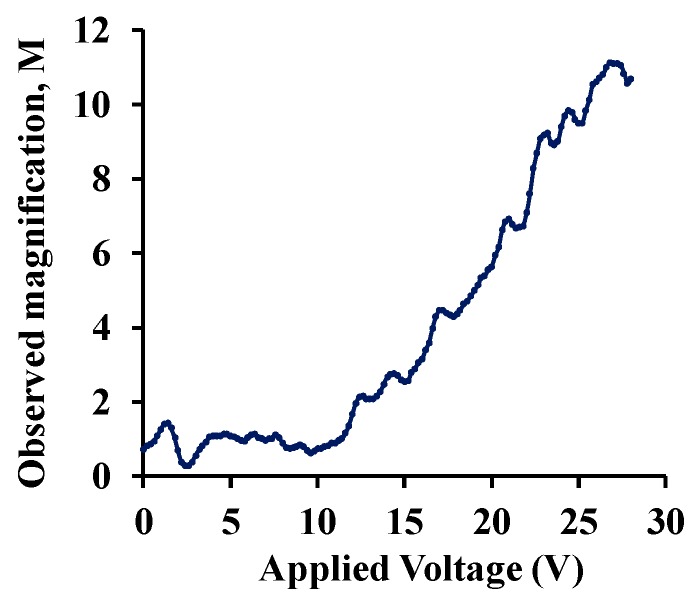
Observed sensor output magnification as a function of applied bias voltage at constant relative humidity.

**Figure 8:**
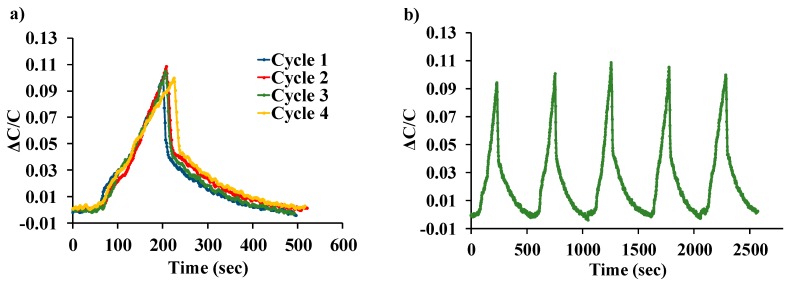
(**a**) Repeatability of LACM sensor tested over four cycles. (**b**) Sensor output over five consecutive cycles of exposure and removal of humidity.

Figure 6a shows the normalized response of the sensor as a function of varying water-vapor concentration at different biasing voltages. Sensor capacitance decreases when operated at no-bias voltage, which we believe is due to a reduction in overlap area because of undesirable out-of-plane downward deflection of the central cantilever beam when exposed to increasing humidity. Application of a small DC bias voltage results in stiffening of the out-of-plane spring constant of the central finger due to induced electrostatic levitation, as described by Tang et al. [49], which prevents out-of-plane deflection. Figure 6b shows the sensor characteristics at different relative humidity levels as a function of varying bias voltage, indicating sensor output amplification as the bias voltage is increased at a constant humidity level. An ~11-fold magnification of sensor response was observed for a bias voltage of 28 V, compared to when a low bias (5 V) voltage was used at 40.83%RH, as shown in Figure 7. The noise floor was experimentally measured to be approximately the same (~400 aF), both with and without parametric amplification corresponding to a noise current of 75.4 pA and dominated by electronic noise in the setup and the capacitance meter. Figure 8 shows the dynamic response of the device when exposed to a gradual change in relative humidity from 20–90%, and operated at a bias voltage of 28 V. Highly repeatable device performance was observed, and no sensor saturation was seen, as shown in Figure 8a. Figure 8b shows the continuous operation of the sensor over five cycles of chamber humidification/dehumidification and shows near zero baseline drift.

### 4.3. Model Fitting

**Figure 9:**
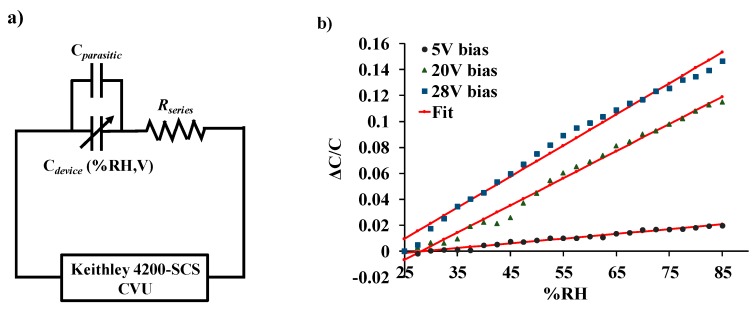
(**a**) Equivalent electrical representation of the LACM sensor. (**b**) Normalized change in capacitance of the sensor curve fitted to Equation (13).

Figure 9a shows the equivalent electrical circuit of the LACM sensor. The humidity response of the device was curve-fitted to Equation (13), and the plot is shown in Figure 9b. Parameter extraction revealed the mean value of *β_p_*~0.114 mN·m^−1^ per ppm of water vapor, and the value of the fitting parameters *A_o_* and *B_o_* ranged from 2.5 to 12 and −0.06 to −0.01, respectively. The root-mean-square error was found to be 0.1%, 0.39%, and, 0.52% for applied bias voltages 5 V, 20 V, and 28 V, respectively.

### 4.4. Absorption-Desorption Kinetics

**Figure 10:**
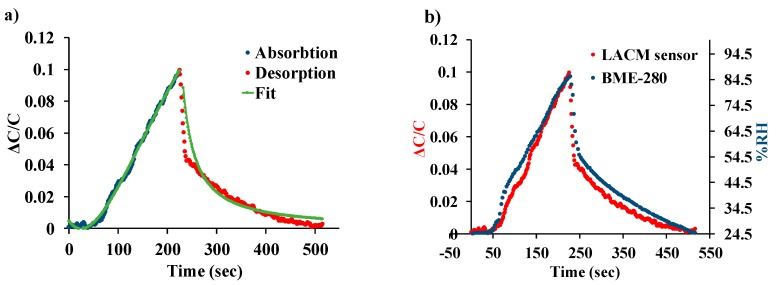
(**a**) Dynamic response of the LACM sensor curve fitted to Fick’s second law and the Polanyi–Wigner equation. (**b**) Comparison of the LACM sensor performance to a commercially available BME-280 reference chip.

The dynamic response of the device is dependent on the moisture absorption induced swelling of the polyimide on the central cantilever beam. This type of behavior can be explained by a modified version of Fick’s second law of diffusion. The performance of the LACM sensor can be effectively modeled as described by Sikame Tagne [50]. The desorption kinetics of the sensor is considered as the gradual desorption of water molecules back into the atmosphere and modeled using the Polanyi–Wigner equation [51]. The curve fitted normalized change in capacitance is shown in Figure 10a when the humidity is increased from 25–85% and then decreased back to 25%. Additionally, Figure 10b compares the normalized device response to that of a commercially available BME-280 [48] reference sensor chip. It can be observed from the plot that the LACM sensor closely follows the response of the reference chip, which has a response time of 1 s [48] with both the sensors reaching their maximum output at the same time.

## 5. Conclusions

We presented the design, fabrication, and response of a batch-fabricated capacitive polymer-based humidity sensor based on mechanical leveraging and parametric amplification. The device exploits the electrostatic lateral instability of MEMS structures to achieve a noiseless intrinsic gain, which helps in achieving a better SNR. An ~11-fold magnification in sensor output was achieved by applying a 28 V DC bias voltage to the device at constant water vapor concentration due to spring softening. We demonstrated an unassisted and completely recoverable change of 11% in capacitance value when subjected to a humidity change from 25–85%. The dynamic response of the sensor was also characterized, and the sensor showed a comparable response to a commercially available reference chip with ~1 s response time. A mathematical model to accurately describe the sensor action and sensor dynamics has also been presented. Such a sensor is an excellent candidate for a low-power, low-cost, and sensitive vapor-sensor for applications in IoT based frameworks. Future work would involve using the LACM sensor for the detection of other analytes, such as volatile organic compounds, and addressing the issue of temperature sensitivity of this device due to its bimorph type structure.
